# A Preliminary Study on Polyurethane Foam Ingestion by *Tenebrio molitor* Larvae and Potential Effects of Depolymerising Bacterial Supplementation

**DOI:** 10.3390/insects17101057

**Published:** 2026-10-13

**Authors:** Guillermo Fondevila, Sara Remón, Violeta C. Sein-Echaluce, Manuel Fondevila

**Affiliations:** 1 Departamento de Producción Animal y Ciencia de los Alimentos, Instituto Agroalimentario de Aragón (IA2), Universidad de Zaragoza-CITA, M. Servet 177, 50013 Zaragoza, Spain; remon@unizar.es (S.R.); mfonde@unizar.es (M.F.); 2 AITIIP Centro Tecnológico, Polígono Industrial Empresarium, Romero 12, La Cartuja Baja, 50720 Zaragoza, Spain; violeta.calvo@aitiip.com

**Keywords:** mealworm, polyurethane, plastic degradation, probiotics

## Abstract

Intake and utilisation of polyurethane foam by *Tenebrio molitor* larvae, and the potential contribution of depolymerising bacteria (*Kocuria rhizophila*, *Priestia megaterium* and *Cupriavidus respiraculi*) used as probiotics, were evaluated. In Experiment 1, larvae were raised without substrate or with polyurethane foam, with fresh carrot provided as a water source. Mortality (23.8%) was unaffected by treatment (*p* > 0.10), and polyurethane disappearance averaged 22.1 mg/larva. Larval growth (*p* = 0.042) and frass output (*p* < 0.001) were higher in polyurethane-fed larvae. In Experiment 2, treatments consisted of polyurethane alone (PU), with wheat bran (PUB), or wheat bran and a mixed bacterial culture (PUBP). Larval growth and total mass production were higher in PUB and PUBP than in PU (*p* < 0.001), although numerical improvements in PUBP compared to PUB were not significant. Polyurethane disappearance did not differ (9.85 mg/larva, as an average), whereas frass production was higher in PUBP than in PU, with intermediate values for PUB (*p* < 0.001). Microscopic examination revealed numerous polyurethane particles in frass, indicating ingestion and fragmentation. However, no direct evidence of polymer biodegradation or microbial activity within the larval gut was obtained. In conclusion, *T. molitor* larvae ingested polyurethane foam independently of the additional substrate supply, although these findings should be interpreted with caution and degradation mechanisms need to be contrasted in further studies.

## Simple Summary

Plastic waste represents a growing environmental challenge, and insects have been proposed as potential biological agents for plastic processing. This study shows that *Tenebrio molitor* larvae were able to survive and ingest polyurethane foam, even when no additional nutrient substrate was provided. However, larval growth was limited under these conditions. The inclusion of wheat bran improved larval performance, while supplementation with a plastic-degrading bacterial culture further supported growth, although it did not increase polyurethane intake. Microscopic observations indicated that polyurethane was ingested and physically fragmented, but chemical biodegradation could not be confirmed. These results indicate the potential interest of *T. molitor* larvae and bacterial supplementation for plastic processing, but further studies are required to clarify the mechanisms involved in chemical biodegradation.

## 1. Introduction

Plastics are widely used materials that can be highly detrimental to ecosystems due to their toxicity to living organisms and their long-term persistence in the environment, requiring efficient recycling alternatives aimed at minimising their ecological impact [1]. A sustainable option involves their biological degradation by bacteria [2,3]. Goveas et al. [4] reported that plastic degradation efficiency by bacterial strains isolated from natural environments ranged from 3 to 17%. In this respect, particularly promising results have been reported for co-cultured bacterial species such as *Priestia megaterium* [5,6] and others belonging to the genera *Kocuria* [7,8] and *Cupriavidus* [8,9].

Several insect species have been proposed as potential plastic recyclers [10,11,12,13]. These insect species can ingest plastics, allowing their mixture with the digestive microbiota and the partial degradation of polymer chains by enzymatic action, resulting in the potential absorption of oligomers as an energy source [14]. In this regard, larvae of the coleopteran species *Tenebrio molitor*, which has been proposed as a sustainable alternative for the production of nutrients for both livestock and humans [15,16,17], have shown a potential degradative activity of plastics such as polystyrene, polyethylene and polypropylene [18,19,20]. Moreover, the ability of *P. megaterium,* isolated from the gut of *T. molitor* larvae, to degrade polystyrene foam has been demonstrated [21]. Urbanek et al. [22] and Orts et al. [23] observed that *T. molitor* larvae were able to reduce the mass of polystyrene and polyurethane foam when provided as substrates. However, these authors observed that larvae lost a substantial part of their initial body weight, suggesting that the energy potentially obtained through plastic depolymerisation may be limited. Other authors have also reported larval weight losses when fed other plastics such as polystyrene, polypropylene, polyvinyl chloride or low-density polyethylene [24,25,26]. Thus, it remains unclear to what extent larvae are able to depolymerise and metabolise each type of plastic, and distinguishing between plastic ingestion, mechanical fragmentation, and true biodegradation remains a major challenge in this field. Previous research [27,28,29] suggested that supplementing the substrate with additional nutrient sources such as wheat bran could improve larval ability to ingest and degrade plastics, thus supporting larval survival. Furthermore, it could be hypothesised that some culturable bacteria fed as probiotics could contribute to depolymerisation if they were able to be established within the digestive tract of the larvae, as it has been previously tested to provide pathogen resistance in *T. molitor* larvae [30,31]. This study aimed to evaluate the extent to which *T. molitor* larvae can ingest polyurethane foam and whether they are capable of degrading it and using it as an energy source when supplemented with a culture of depolymerising bacteria (*K. rhizophila*, *P. megaterium* and *C. respiraculi*) acting as probiotics in order to assess the potential suitability of this strategy to reduce the ecological impact of plastics.

## 2. Materials and Methods

### 2.1. Experiment 1

*T. molitor* larvae at approximately 8 weeks after hatching, previously fed on wheat bran, and measuring 10 to 12 mm in length were obtained from our colony and used for the experiment. A total of 480 larvae (25.75 ± 4.73 mg) were separated and collected by sieving, manually sorted, and distributed into homogeneous groups (*n* = 60) in 8 plastic trays (15 × 9 × 6 cm). Two experimental treatments were tested: the absence of substrate (NS) or 2.05 ± 0.049 g of polyurethane foam as the only dry substrate (PU), in order to evaluate larval survival and the ability of larvae to consume this plastic. A commercial semi-rigid polyurethane foam supplied to AITIIP Centro Tecnológico was used. The material presented an apparent density of 40 kg/m^3^ and a closed-cell structure. The dry matter (DM) content of the polyurethane foam was 99.12%. In addition, two pieces of fresh carrot (9.1% DM) were added to all trays twice a week as a source of water, vitamins and minerals (3.43 ± 0.050 g fresh matter per week per tray). The trays were incubated for 49 days in a climatic chamber (CPR 240 Premium, VWR International Eurolab, Barcelona, Spain), maintained at 27 °C and 60% relative humidity, and kept in darkness except during sampling.

Total larval mass and the number of living larvae per tray were recorded weekly to estimate average larval weight and mortality. From these data, larval growth was calculated as the difference between the average initial and final weights. Polyurethane foam residues per tray were weighed at the end of the experimental period and total polyurethane disappearance was calculated. When polyurethane disappearance was expressed per larva, the value was corrected by the survival-weighted mean, calculated as the average number of larvae present in each tray throughout the experimental period. At the end of the experiment, larvae were killed by freezing at −80 °C and subsequently lyophilised to determine DM content. Total frass produced per tray was collected, weighed and dried (105 °C for 24 h) to determine DM content.

### 2.2. Experiment 2

*T. molitor* larvae (25.30 ± 3.47 mg) were collected as indicated for Experiment 1 and distributed in groups of 60 into 18 trays containing 3.62 ± 0.082 g of polyurethane foam. Three experimental treatments were defined: polyurethane as the only dry substrate (PU), polyurethane supplemented with wheat bran (PUB) and polyurethane supplemented with both wheat bran and a bacterial culture used as a probiotic (PUBP). The amount of wheat bran (% fresh matter: DM, 89.2; crude protein, 13.8; starch, 35.8; neutral detergent fibre, 55.5) added was estimated as 40% of total intake of larvae reared on wheat bran fed *ad libitum* as the sole feed in previous experiments [32] and was supplied in two equal weekly doses of 0.20 g per tray. The bacterial probiotic consisted of a mixed culture of *Kokuria rhizophila* (2.82 × 10^5^ colony-forming units per gram of agar, cfu/g)*, Priestia megaterium* (4.81 × 10^4^ cfu/g) and *Cupriavidus respiraculi* (1.15 × 10^5^ cfu/g), grown on agar. Bacterial strains isolated from a plastic waste dump were provided by CETEM (Centro Tecnológico del Mueble y la Madera, Murcia, Spain), and were identified by matrix-assisted laser desorption/ionization time-of-flight mass spectrometry (MALDI-TOF MS) using a Microflex L20 (Bruker Daltonics, Fremont, CA, USA) equipped with an N_2_ laser, applying the ‘extended direct transfer’ method. As described by Muñoz-Martí et al. [33], spectra were acquired in positive linear ion mode with an acceleration voltage of 20 kV. Each spectrum corresponded to the sum of 240 shots per target, with a mass range of 2000–20,000 Da, and three spectra per strain were obtained. Identification was performed using the MALDI Biotyper Realtime Classification method against reference databases, including MBT 7854 (Bruker Daltonics), MBT 7311_RUO (Bruker Daltonics), the MALDI-TOF database of the Spanish Type Culture Collection (CECT), and the Drinking Water Library (DWL). After identification, the depolymerising capacity of these strains was assessed as described by Muñoz-Martí et al. [33], based on absorbance measurements after 14 days of culture. All bacterial strains were stored in glycerol stocks at −80 °C until use. For revitalisation, bacteria were inoculated in liquid nutrient broth (NB; Liofilchem, Roseto degli Abruzzi, Italy) until approximately 10^7^ cfu/mL. Then, 50 µL aliquots were streaked onto nutrient agar plates (Liofilchem, Roseto degli Abruzzi, Italy). Plates were incubated for 24 h at 33 °C and stored at 4 °C until use. Two agar pieces equivalent to 0.24 mL each (approximately 4.8 mg agar) were provided twice weekly to the trays assigned to the PUBP treatment, providing a regular source of bacterial inoculum throughout the experimental period. All trays received 3.46 ± 0.098 g fresh carrot (10.7% DM) per tray, twice a week as a source of water, vitamins and minerals. An example of the incubated trays illustrating the incubation conditions is shown in Figure 1. Trays were maintained for 28 days at 27 °C and 60% relative humidity in darkness throughout the experiment.

Larval mortality was estimated weekly as indicated for Experiment 1. Larvae per tray and total frass produced were weighed at the end of the experimental period and average larval weight, larval growth, and frass production were calculated. Substrate residues (polyurethane, wheat bran and fresh carrot, according to treatment) per tray were weighed at the end of the experimental period and total substrate disappearance was estimated on a DM basis. Polyurethane particles recovered in the frass were considered ingested material, as they had necessarily passed through the digestive tract, and were therefore not included in the residual fraction. The feed to gain ratio (F:G) was calculated as the amount of total feed substrate consumed (polyurethane, wheat bran and carrot) minus the remaining residue after 28 days per unit of larval mass gained. All the fresh carrot and wheat bran offered were completely consumed in all cases, and polyurethane disappearance per larva was calculated as indicated for Experiment 1. For the assessment of polyurethane excretion, representative samples of frass (25 mg) from each tray were hydrolysed with 4 mL of 30% H_2_O_2_ at 50 °C for 24 h and filtered through a Durapore PVDF membrane filter with a pore size of 5 µm (Merck Millipore, Burlington, MA, USA). Filtered samples were examined under a microscope (Axiolab, Carl Zeiss Jena, Germany) using a 10× eyepiece and a 10× objective.

### 2.3. Statistical Analysis

Data from Experiment 1 (NS vs. PU) and Experiment 2 (PU vs. PUB vs. PUBP) were analysed statistically by ANOVA, with the experimental treatment as the main source of variation, using the GLM procedure of SAS 15.1 [34]. Data were tested for normality using the Shapiro–Wilk test prior to analysis. In all cases, the tray was considered as the experimental unit (*n* = 4 in Experiment 1 and *n* = 6 in Experiment 2). Weekly patterns of larval body weight and mortality were analysed using the MIXED procedure of SAS, considering sampling date as a repeated factor. The tray was considered the experimental unit. Four covariance structures (compound symmetry and first-order autoregressive models, each assuming either homogeneous or heterogeneous variances) were compared. The model assuming compound symmetry with heterogeneous variances was selected according to the lowest AIC and BIC values. When treatment effects were significant, mean differences were compared using Tukey’s test. Differences with *p* < 0.05 were considered significant.

## 3. Results

### 3.1. Experiment 1

The effects of polyurethane foam supplementation on growth performance and frass production of *T. molitor* larvae from 0 to 49 days of Experiment 1 are presented in Table 1. Total mortality rates were high (23.75%, as an average) and no differences between treatments were detected (*p* > 0.10) at any of the experimental times considered (Figure 2). Final larval weight and DM content did not differ between treatments but larval growth was higher for PU than for NS (*p* = 0.040). In fact, total larval mass produced per tray, both expressed on a fresh matter (*p* = 0.023) and DM (*p* = 0.028) basis, increased in larvae fed PU compared to those fed NS. Larvae consumed the entire supply of fresh carrot offered throughout the experiment in all cases (21.99 and 21.65 g DM/tray for PU and CT, respectively), and polyurethane foam disappearance in PU was 1.18 ± 0.23 g DM/tray, averaging 21.93 mg DM/larva. Frass production was higher for PU than for CT, both on a fresh matter and DM basis (*p* < 0.001).

### 3.2. Experiment 2

The effects of treatment on growth performance of *T. molitor* larvae from 0 to 28 days of Experiment 2 are presented in Table 2. Total mortality averaged 7.19% and no treatment effects were observed throughout the experiment. Final larval weight was higher for larvae fed PUBP compared to those fed PU, with larvae fed PUB showing intermediate values (*p* = 0.017). Larval growth and DM content, as well as total larval mass per tray, expressed both on fresh matter and DM basis, were higher in PUBP and PUB than in PU (*p* < 0.001). In addition, F:G ratio was poorer for PU than for PUB and PUBP (*p* < 0.001). No differences among treatments were recorded for polyurethane foam disappearance (0.560 g DM/tray and 9.85 mg DM/larva, as an average) from 0 to 28 days of experiment (Table 3). Total fresh carrot supplied to all the experimental groups throughout the experimental period (1.48 g DM/tray, as an average) was completely consumed. Similarly, larvae from treatments PUB and PUBP consumed the entire supply of wheat bran supplied (1.47 g DM/tray, as an average) in all cases. As a result, total DM intake was lower for PU than for PUB and PUP (*p* < 0.001). Frass production was highest for PUP, intermediate in PUB, and lowest in PU0 (*p* < 0.001).

For the assessment of polyurethane excretion, the high number of polyurethane particles, together with their irregular shapes and wide variability in size (ranging from 120 to 290 µm in length and 40 to 210 µm in width), made it impossible to quantify polyurethane content in the frass by microscopic evaluation. Figure 3 shows representative images of hydrolysed frass samples from the three treatments.

## 4. Discussion

The potential ability of some insect species to consume plastics has been attributed to the relatively high microbial activity involved in the degradation of plastic polymer chains within the insect gut microbiota, highlighting its relevance in plastic biotransformation [18,22,30]. In addition, the presence of polystyrene-degrading enzymes has been reported in the saliva of certain insect species such as *Galleria mellonella* larvae [12,13]. Nevertheless, the nutritional value of plastics as an energy source is likely to be low. In this respect, previous studies showed larval weight losses when plastics were provided as the sole substrate, although mortality rates were ultimately similar to those observed with conventional substrates [24,25]. Previous research from our group indicated that mortality rates in *T. molitor* larvae fed nutrient-rich substrates range from 5 to 10% [32,35]. In this respect, the results observed in Experiment 1 showed a high larval mortality (23.8%, as an average) when nutrient availability was restricted in both the NS and PU groups, but comparable [19,36] or lower [37] than values previously reported for other plastics. In addition, observations from Experiment 1 suggested a high variability in larval size within each tray. This variability was probably the result of both the limited availability of nutrients and the need to adapt to polyurethane as a feeding substrate, which may have promoted competitive behaviour and heterogeneous levels of consumption among larvae, resulting in a lack of uniformity in larval development. Nevertheless, these observations were not quantitatively assessed and should therefore be interpreted with caution. In any case, the results obtained in Experiment 1 after 49 days of restricted nutrient availability indicate a relatively high resilience capacity of *T. molitor* larvae to survive under adverse conditions of nutrient deprivation.

Other than polymer chains, polyurethane foam contains no components that could be considered nutrients (i.e., amino acids, vitamins, minerals) for the larvae. In Experiment 1, larvae consumed appreciable amounts of polyurethane foam (22.1 mg/larva) in the absence of nutrients other than the limited amounts of minerals, protein and vitamins provided by fresh carrot [38], which was supplied as a water source. This resulted in an approximately two-fold increase in frass production and an appreciable improvement in growth performance by the supplementation of polyurethane for *ad libitum* larval consumption. However, these results should be interpreted with caution, as fresh carrot supplementation may have contributed to larval growth to some extent, potentially masking the response to polyurethane ingestion. Nevertheless, the results obtained indicate that *T. molitor* larvae are able to ingest polyurethane foam. The higher larval biomass production per tray may be consistent with a possible contribution of polyurethane-derived compounds to larval energy metabolism, despite the limited availability of dietary protein to support growth. However, this hypothesis could not be confirmed under the present experimental conditions. These findings further support the potential role of *T. molitor* in the physical processing of this plastic.

Considering the absence of protein in polyurethane, it could be hypothesized that larval growth in Experiment 1 might have been greater if an additional protein source had been provided. Previous research reported that plastic intake by *T. molitor* larvae was increased when polypropylene, polystyrene or polyethylene were co-fed with wheat bran [29,36]. In Experiment 2, larvae reared on polyurethane foam showed increased growth performance and DM content and an improved F:G ratio when supplemented with wheat bran at low doses, calculated to supply a limited but sufficient amount of amino acids to stimulate protein synthesis. However, wheat bran supplementation did not affect polyurethane foam consumption throughout the 28-day experimental period. In this respect, it should be noted that the wheat bran-to-polyurethane foam ratio used in the present study (3:1) was lower than that evaluated in previous research, in which a ratio of 7:1 was reported to maximise polystyrene and polyethylene degradation [36]. Reported values for daily polyurethane intake by *T. molitor* larvae vary widely in the literature depending on the chemical type of polyurethane, larval size, and exposure duration, ranging from 0.07–0.09 mg/day [11,36] to 0.18 mg/day [39] per larva. Calculated daily intake per larva based on values obtained in Experiments 1 and 2 averaged 0.45 and 0.35 mg/larva, respectively, which are well above previously reported values. This difference may be affected by the additional supplementation of fresh carrot as source of water, which could stimulate polyurethane consumption. However, the results obtained do not support an improvement in larval activity related to polyurethane foam intake as a response of the inclusion of an additional nutrient source such as wheat bran, highlighting that ingestion rates were apparently independent of nutrient supplementation under the current experimental conditions.

Several bacterial species have been reported to exhibit depolymerising activity against different types of plastics, and their use is considered as a potentially sustainable option to reduce plastic contamination [40,41]. In this context, Muñoz-Martí et al. [33] reported that selected bacterial strains isolated from different environments exhibited effective polyurethane foam degradation activity in biodegradation assays, with values of 59, 36 and 88% for *K. rhizophila*, *P. megaterium* and *C. respiraculi*, respectively. The presence of *P. megaterium* and *Kocuria* spp. in isolates from the gut of *T. molitor* and *Alphitobius diaperinus* larvae has been previously confirmed [21,42]. However, to our knowledge, the ability of *C. respiraculi* to survive and establish in the gut of these insects has not been previously evaluated. The previous detection of these microorganisms in the gut of the larvae further suggests a potential compatibility with the intestinal environment and provided part of the rationale for evaluating these strains as probiotic candidates in the present study. However, their persistence, metabolic activity and contribution to polymer transformation under in vivo conditions remain largely unexplored. In Experiment 2, the numerical improvement in larval growth and F:G ratio observed in larvae from the treatment supplemented with the bacterial probiotic culture (PUBP) compared to the PUB group did not reach significance. Consequently, it could not be demonstrated that the selected bacterial species were able to influence responses associated with polyurethane foam utilisation by the larvae. In addition, it should be acknowledged that the bacterial inoculum was administered on agar, which was not supplied to the PUB treatment. Given the very small amount of agar provided and its limited nutritional value, its influence on the comparison between PUB and PUBP treatments was likely minimal, although it cannot be completely ruled out. On the one hand, it should be considered that the nutritional value of plastic-derived compounds, if released and absorbed after passage through the digestive tract, is expected to be low, and their impact on larval performance may not be enough to induce a significant response. Also, polymer degradation by bacteria often requires long exposure times [43] and consequently, the relatively fast passage rates of the digesta throughout the gastrointestinal tract of the larvae might potentially limit polyurethane foam utilisation. In addition, discrepancies between the optimal growth conditions of the supplemented bacteria and the physicochemical environment of the larval gut (i.e., oxygen availability) may have further constrained their activity. On the other hand, bacterial establishment within the gastrointestinal tract of the larvae was not assessed in this study, and the absence of information regarding the gut microbial community limits the interpretation of the effects of bacterial supplementation. In this respect, the cultured microorganisms used in this study grow under aerobic conditions (*K. rhizophila* and *C. respiraculi*) or are facultative anaerobes (*P. megaterium*), whereas the environment within the larval digestive tract is generally anaerobic. Therefore, it could not be confirmed whether the depolymerising activity of the selected species was insufficient to promote a marked improvement in polyurethane foam utilisation, or whether these bacteria were not able to establish within the larval gut. However, the results observed in the current study suggest that the possibility that these bacterial species may establish in the digestive tract of *T. molitor* larvae cannot be completely excluded, although this remains to be demonstrated under controlled experimental conditions.

Bulak et al. [11] recovered 42 to 45% of the ingested plastic mass in the frass of *T. molitor* larvae fed two different types of polyurethane. Similarly, Peng et al. [19] and Yang et al. [24] reported 35 and 49% plastic excretion when larvae were fed polyvinyl chloride and polystyrene foam, respectively. The original plan of Experiment 2 was to quantitatively evaluate polyurethane excretion under microscope examination of frass samples after H_2_O_2_ hydrolysis. In this respect, residual polyurethane foam pieces in the frass were easily separated from the excreta, despite the likely generation of small fragments and microplastics through chewing. Unfortunately, the limited amount of sample available, together with the irregular shapes and wide variability in size of polyurethane particles in the frass, did not allow a proper quantification of polyurethane excretion by microscopic evaluation. Nevertheless, qualitative observations among treatments suggested that frass samples from the PUB and PUBP groups contained fewer polyurethane particles, which also appeared to be smaller in size, than those observed in samples collected from larvae fed PU, although this difference could not be quantified. Therefore, the results of this study confirm the ability of *T. molitor* larvae to ingest polyurethane, but it could not be elucidated whether larvae are capable of digesting and absorbing this material, acting as effective plastic degraders under the experimental conditions evaluated, and in practical situations it is important to distinguish between ingestion, mechanical fragmentation, and true biodegradation, although these processes are often confounded in the literature. In this respect, the challenge associated with plastic biodegradation cannot be addressed solely by reducing particle size through mechanical fragmentation, as microplastics may pose greater environmental risks than larger plastic particles [1,4]. Effective mitigation of plastic pollution therefore requires true biotransformation into metabolically available compounds.

## 5. Conclusions

The results indicate that *T. molitor* larvae are able to survive under conditions of limited nutrient availability. In this context, larvae were able to ingest appreciable amounts of polyurethane foam in the absence of other conventional feed substrates, resulting in abundant excretion of plastic particles and only slight increases in larval growth, which do not provide conclusive evidence that polyurethane-derived compounds were metabolically utilised by the larvae. However, the lack of chemical characterisation of polyurethane before and after ingestion (e.g., FTIR, GPC or GC-MS analyses) represents a limitation of the present study. Such approaches would be required to confirm structural modifications of the polymer and to distinguish between mechanical fragmentation and actual polymer biodegradation. From an ecological perspective, these micro- and nanoplastic particles may represent a relevant environmental concern and consequently, effective strategies to biodegrade these compounds are required to promote their complete removal from the environment. Supplementation with low amounts of wheat bran in larvae fed polyurethane foam enhanced larval growth and improved the F:G ratio, without affecting plastic intake. These results do not support the hypothesis that the capacity of larvae to consume plastics and act as biological processing agents can be substantially enhanced through co-feeding with an additional nutritional source. Finally, supplementation with a mixed culture of polyurethane-degrading microorganisms (*K. rhizophila*, *P. megaterium* and *C. respiraculi*) used as probiotics did not result in significant improvements in larval growth performance or total plastic consumption. This lack of response may be related to the low nutritional value of the substrate and the limited impact of any potential increases in the digestibility and utilisation of plastic-derived compounds. However, the numerical improvements observed in productive performance in probiotic-supplemented larvae suggest that a potential effect of bacterial supplementation cannot be completely excluded. In this regard, the potential role of this strategy should be further evaluated in future studies, with particular emphasis on quantifying total plastic excretion and tissue retention, as well as confirming the establishment and activity of the most suitable supplemented bacteria within the larval gastrointestinal tract. These issues should be complemented by the use of analytical techniques to assess polymer transformation, which were not assessed under the experimental conditions of the present study.

## Figures and Tables

**Figure 1:**
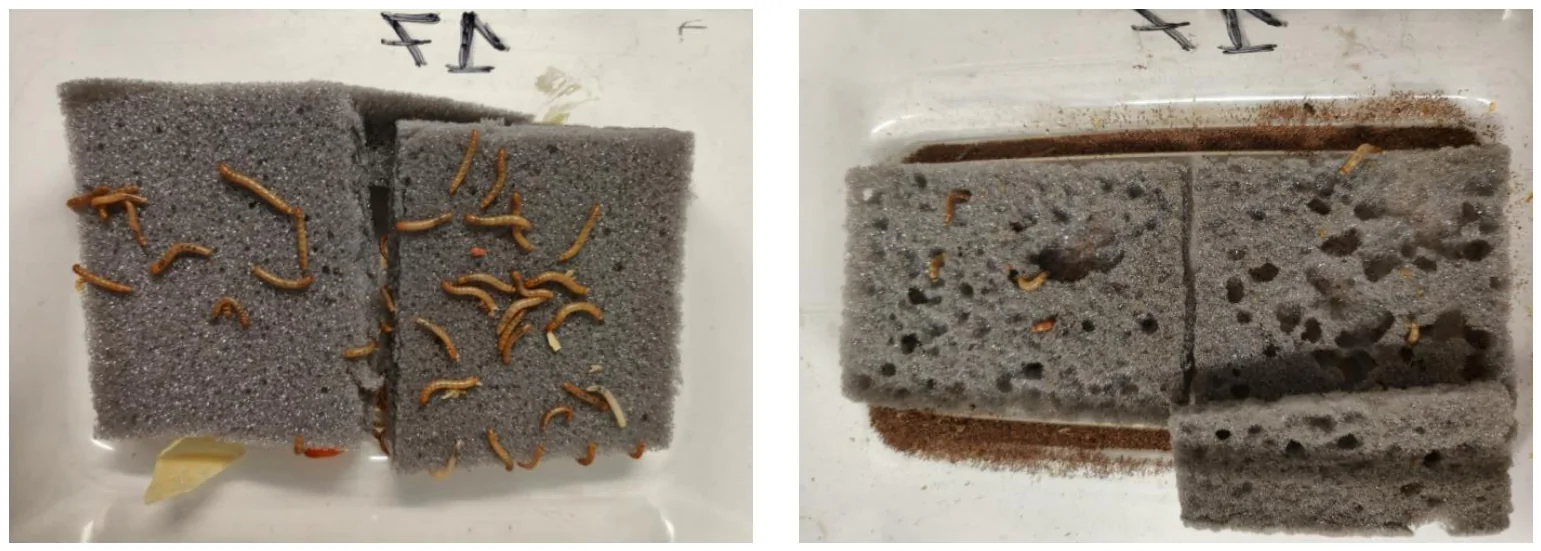
Representative images of incubation trays containing *T. molitor* larvae at week 0 (**left**) and week 4 (**right**), in Experiment 2.

**Figure 2:**
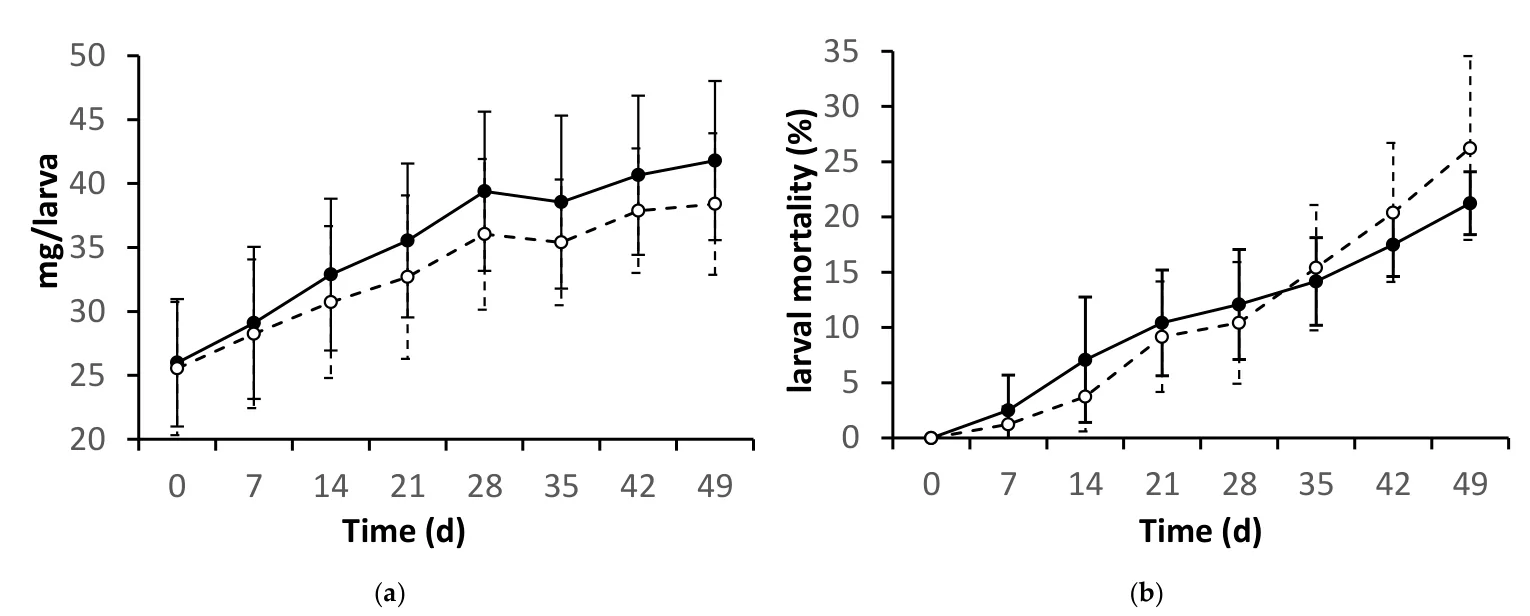
Weekly pattern of average weight (**a**) and cumulated larval mortality (**b**) of *T. molitor* larvae fed fresh carrot with (PU, black circles) or without (NS, white circles) polyurethane foam over 49 days (Experiment 1). No treatment differences on larval mortality or average weight were detected at any time considered (*p* > 0.10).

**Figure 3:**
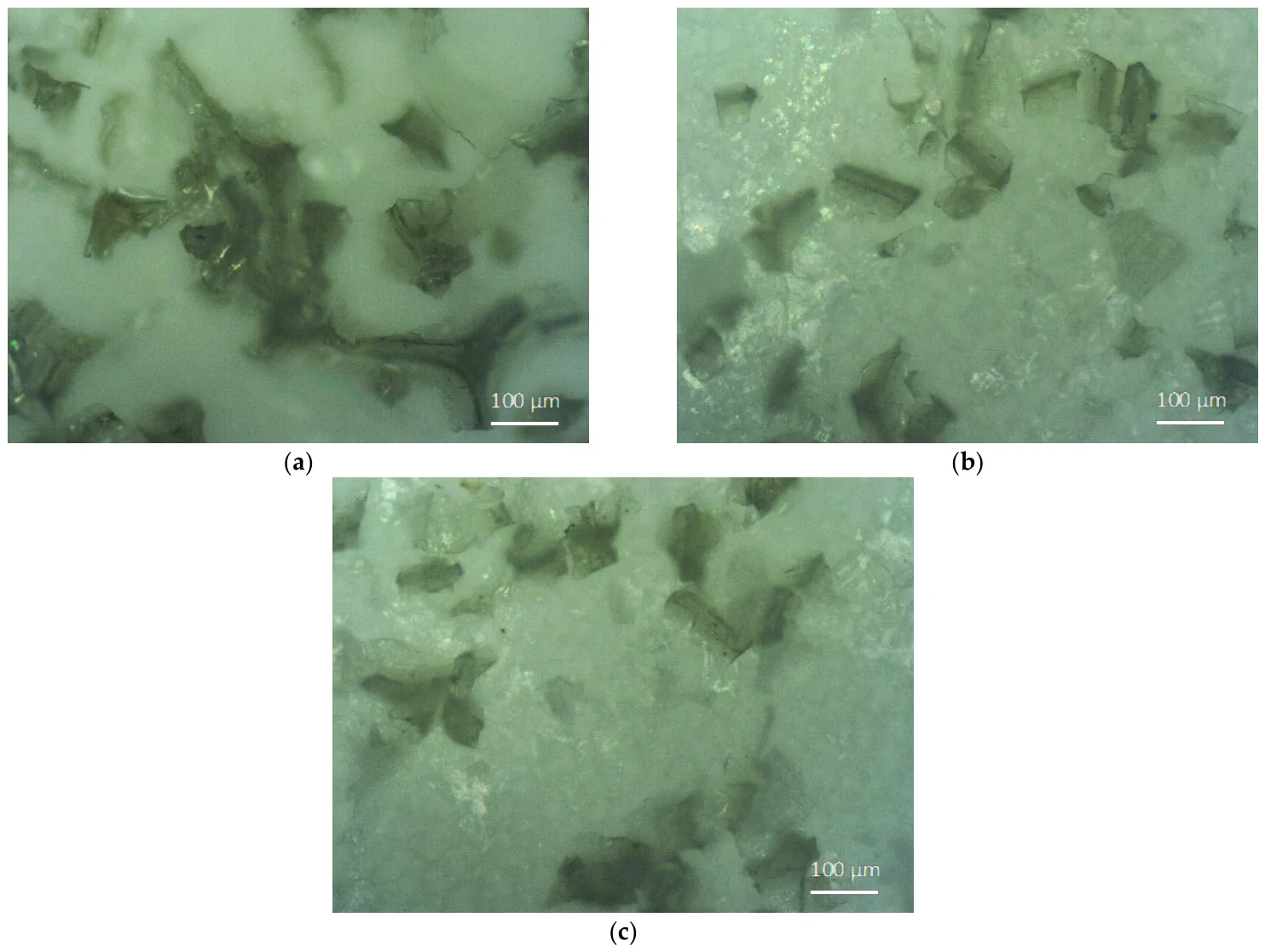
Representative images of polyurethane particles excreted by *T. molitor* larvae fed polyurethane foam (PU, (**a**)) or polyurethane foam supplemented with wheat bran, without (PUB, (**b**)) or with probiotics (PUBP, (**c**)) over 28 days (Experiment 2).

**Table 1:** Growth performance and frass production of *T. molitor* larvae fed fresh carrot with (PU) or without (NS) polyurethane foam over 49 days ^1^ (Experiment 1).

	PU	NS	SEM (*n* = 4)	*p*-Value
Final mortality ^2^, %	21.3	26.3	3.11	0.299
Initial larval weight, mg	26.0	25.5	2.55	0.894
Final larval weight, mg	41.8	38.4	2.95	0.445
Larvae, % dry matter	28.7	26.3	1.30	0.234
Larval growth, mg	15.8	12.9	0.79	0.040
Larval mass, g/tray				
Fresh matter	0.423	0.180	0.0566	0.023
Dry matter	0.110	0.050	0.0150	0.028
Frass production, g/tray				
Fresh matter	2.28	1.08	0.065	<0.001
Dry matter	2.13	0.98	0.068	<0.001

SEM: standard error of means; ^1^ Larval intake per tray (dry matter basis) was 2.18 ± 0.03 g of carrot (PU and NS) and 1.18 ± 0.21 g of polyurethane foam (PU); ^2^ No differences between treatments were detected for the weekly pattern of larval mortality throughout the experiment.

**Table 2:** Growth performance of *T. molitor* larvae fed polyurethane foam (PU) or polyurethane foam supplemented with wheat bran, without (PUB) or with probiotics (PUBP) over 28 days ^1^ (Experiment 2).

	PU	PUB	PUBP	SEM (*n* = 6)	*p*-Value
Final mortality ^2^, %	7.78	7.39	6.39	2.210	0.901
Initial larval weight, mg	25.3	25.2	25.3	1.51	0.998
Final larval weight, mg	39.8 ^b^	48.2 ^ab^	50.3 ^a^	2.37	0.017
Larvae, % dry matter	22.3 ^b^	27.2 ^a^	27.5 ^a^	0.45	<0.001
Larval growth, mg	14.5 ^b^	22.9 ^a^	24.9 ^a^	1.10	<0.001
Larval mass, g/tray					
Fresh matter	0.677 ^b^	1.147 ^a^	1.293 ^a^	0.0602	<0.001
Dry matter	0.149 ^b^	0.312 ^a^	0.355 ^a^	0.0136	<0.001
F:G, mg/mg (DM basis)	14.12 ^a^	11.32 ^b^	9.93 ^b^	0.646	<0.001

SEM: standard error of means; F:G: feed to gain ratio; DM: dry matter. Within rows, letters indicate treatment differences (*p* < 0.05). ^1^ All trays were provided with polyurethane foam for *ad libitum* consumption. ^2^ No differences between treatments were detected for the weekly pattern of larval mortality throughout the experiment.

**Table 3:** Substrate intake and frass production per tray of *T. molitor* larvae fed polyurethane foam (PU) or polyurethane foam supplemented with wheat bran, without (PUB) or with probiotics (PUBP) over 28 days ^1^ (Experiment 2).

	PU	PUB	PUBP	SEM (*n* = 6)	*p*-Value
Polyurethane disappearance, g	0.583	0.548	0.548	0.0337	0.703
Total intake ^2^, g (DM basis)	2.07 ^b^	3.48 ^a^	3.50 ^a^	0.033	<0.001
Frass production, g					
Fresh matter	1.13 ^c^	1.95 ^b^	2.24 ^a^	0.035	<0.001
Dry matter	1.04 ^c^	1.81 ^b^	2.09 ^a^	0.033	<0.001

SEM: standard error of means; DM: dry matter. Within rows, letters indicate treatment differences (*p* < 0.05). ^1^ All trays were provided with polyurethane foam for *ad libitum* consumption. ^2^ Sum of polyurethane foam, fresh carrot and wheat bran consumption according to treatment. Larval intake per tray (DM basis) was 1.48 ± 0.04 g of carrot (PU, PUB, and PUBP) and 1.47 ± 0.04 g of wheat bran (PUB and PUBP).

## Data Availability

The original contributions presented in this study are included in the article. Further inquiries can be directed to the corresponding author.

## Abbreviations

The following abbreviations are used in this manuscript:

cfu	Colony-forming units
DM	Dry Matter
F:G	Feed to Gain ratio
SEM	Standard error of means
